# A troglobitic species of the centipede *Cryptops* (Chilopoda, Scolopendromorpha) from northwestern Botswana

**DOI:** 10.3897/zookeys.977.57088

**Published:** 2020-10-22

**Authors:** Gregory D. Edgecombe, Nesrine Akkari, Edward C. Netherlands, Gerhard Du Preez

**Affiliations:** 1 The Natural History Museum, Cromwell Road, London SW7 5BD, UK The Natural History Museum London United Kingdom; 2 Naturhistorisches Museum Wien, Burgring 7, 1010 Wien, Austria Naturhistorisches Museum Wien Wien Austria; 3 Unit for Environmental Sciences and Management, North-West University, Private Bag X6001, Potchefstroom 2520, South Africa North-West University Potchefstroom South Africa

**Keywords:** biospeleology, Cryptopidae, molecular phylogenetics

## Abstract

A new species of *Cryptops*, C. (Cryptops) legagus**sp. nov.**, occurs in caves in the Koanaka and Gcwihaba Hills in northwestern Botswana. Bayesian molecular phylogenetics using 18S rRNA, 28S rRNA, 16S rRNA and cytochrome *c* oxidase subunit I corroborates a morphological assignment to the subgenus Cryptops and closest affinities to southern temperate species in South Africa, Australia and New Zealand. The new species is not conspicuously modified as a troglomorph.

## Introduction

*Cryptops* Leach, 1815 is one of the most speciose and geographically widespread centipede genera. Its 150+ species are mostly epigean, but also include troglomorphic species. Troglomorphs display typical modifications of cavernicolous centipedes in general, such as elongation of the antennae, legs and body, and some degree of depigmentation. Compared to epigean species, troglomorphic *Cryptops* usually have an increased number of tibial and tarsal saw teeth (a diagnostic character of the genus) associated with the elongate articles of the ultimate leg pair.

Troglomorphic species of *Cryptops* have been documented from scattered parts of the world. They include endemic species of the subgenus Cryptops from France (Matic 1960), the Canary Islands (Zapparoli 1990), and Brazil (Ázara and Ferreira 2014), and of the subgenus Trigonocryptops Verhoeff, 1906, from Spain (Ribaut 1915), Cuba (Matic et al. 1977), Australia (Edgecombe 2005, 2006), and Brazil (Ázara and Ferreira 2013). Several additional species collected from caves are epigean in most occurrences (Negrea 1993; Stoev 2001). A few other species, including records from Greece, Kenya, India, and Morocco, have been collected only from caves but do not depict troglomorphic characters (reviewed by Edgecombe 2005; also Stavropoulos and Matic 1990).

Herein we add to geographic coverage of troglobitic *Cryptops* by documenting a new species from caves in the Koanaka and Gcwihaba Hills in Ngamiland, northwestern Botswana.

## Habitat

*Cryptops legagus* sp. nov. was collected from Diviner’s (20°8'32.20"S, 21°12'36.60"E) and Dimapo (20°1'12.34"S, 21°21'38.41"E) caves, which are associated with the Koanaka and Gcwihaba Hills, respectively, in Ngamiland, Botswana. These hills, located 20 km apart, are composed of Precambrian dolomites from the Damara Sequence (Williams et al. 2012). Diviner’s and Dimapo caves were discovered by means of gravimetric surveys and exploration drilling followed by the sinking of vertical shafts (70–100 cm diameter). No known natural openings exist. As a result of being sealed, the environmental conditions in these caves are very different from those of other caves with natural openings found on the same hills (Du Preez et al. 2015). Using a Fluke 971 meter, the average temperature and relative humidity levels in Diviner’s Cave were 28.5 ± 0.5 °C and 93 ± 5.4%, respectively, as measured on 12 January 2016. Du Preez et al. (2015) reported similar temperature (maximum of 28 °C), but higher relative humidity (maximum 99.9%) levels in Dimapo Cave. Basic measurements in caves with natural openings from the same region recorded average temperature and relative humidity levels of 18 °C and 93%, respectively, during the hot summer months.

The type locality is Paradise Road Balcony, a sampling site within Diviner’s Cave at which a single specimen (the holotype) was found dwelling in the cave sediment substrate and fig roots associated with the cave floor. Other invertebrates were also collected from this site, including the pseudoscorpion *Botswanoncus ellisi* Harvey and Du Preez, 2014. Two paratypes were collected from Calcite Baboon Chamber in Diviner’s Cave and were primarily associated with large fig tree roots that penetrate the cave roof [see Harvey and Du Preez (2014) for an optical image of the root system]. Paratype NHMW 10152 was collected from Pirates Cove, a site associated with Dimapo Cave. This single specimen was found inhabiting old termite structures associated with the cave floor. All specimens were collected at an average depth of 50 metres below surface.

## Materials and methods

### Morphology

Specimens were collected by hand and preserved in 70% ethanol. Types were photographed using a Nikon DS-Ri2 camera mounted on a Nikon SMZ25 stereomicroscope using NIS-Elements Microscope Imaging Software with an Extended Depth of Focus (EDF) patch. Images were edited with Adobe Photoshop CS6 and assembled in InDesign CS6.

Morphological terminology in descriptions follows recommendations by Bonato et al. (2010).

Type material is housed in the Naturhistorisches Museum Wien (prefix NHMW).

### Molecular phylogenetics

A specimen from Diviner’s Cave fixed in 70% ethanol was used for DNA sequencing. Genomic DNA was extracted using the KAPA Express Extract Kit (Kapa Biosystems, Cape Town, South Africa) as per the manufacturer’s instructions. Polymerase chain reaction (PCR) amplifications were performed in a total volume of 25 µL, with 12.5 µL Thermo Scientific DreamTaq PCR master mix (2×) (2× DreamTaq buffer, 0.4 mM of each dNTP, and 4 mM MgCl2), 1.25 μl of each primer (10mM concentration), and 1 μl DNA. The final reaction volume was made up with Milli-q water.

Molecular markers included two nuclear ribosomal genes (18S rRNA and 28S rRNA) and two mitochondrial markers, one ribosomal (16S rRNA) and one protein-encoding (cytochrome *c* oxidase subunit I) following Boyer et al. (2007). The nuclear ribosomal genes were amplified in three overlapping fragments, the 18S rRNA gene was amplified using primer pairs 1F (5'-TACCTGGTTGATCCTGCCAGTAG-3') and 5R (5'-CTTGGCAAATGCTITCGC-3'); 3F (5'-GTTCGATTCCGGAGAGGGA-3') and 18Sbi (5'-GAGTCTCGTTCGTTATCGGA-3'); and 18Sa2.0 (5'-ATGGTTGCAAAGCTGAAAC-3') and 9R (5'-GATCCTTCCGCAGGTTCACCTAC-3') (Giribet et al 1996; Whiting et al. 1997). The fragments of the 28S rRNA gene were amplified using the primer sets 28SD1F (5'-GGGACTACCCCCTGAATTTAAGCAT-3’) and 28Sb (5'-TCGGAAGGAACCAGCTAC-3') (Park and Foighil 2000; Edgecombe and Giribet 2006); 28Sa (5'-GACCCGTCTTGAAACACGGA-3') and 28Srd5b (5'-CCACAGCGCCAGTTCTGCTTAC-3') (Whiting et al. 1997; Schwendinger and Giribet 2005); and 28S4.8a (5'-ACCTATTCTCAAACTTTAAATGG-3') and 28S7bi (5'-GACTTCCCTTACCTACAT-3’) (Schwendinger and Giribet 2005). A fragment of the 16S rRNA gene was amplified using the primer pair 16Sar (5'-CGCCTGTTTATCAAAAACAT-3') and 16Sb (5'-CTCCGGTTTGAACTCAGATCA-3') (Xiong and Kocher 1991; Edgecombe et al. 2002). For COI, a fragment of the gene was amplified using the primer set LCO1490 (5’-GGTCAACAAATCATAAAGATATTGG-3’) and HCO2198 (5’-TAAACTTCAGGGTGACCAAAAAATCA-3’) (Folmer et al. 1994).

For PCR amplification the following conditions were used: initial denaturation at 95 °C for 5 min, followed by 35 cycles, entailing 95 °C denaturation for 30 s, annealing between 45–50 °C for 30 s with an end extension at 72 °C for 1 min, and following the cycles a final extension of 72 °C for 10 min. The PCR reactions were carried out using a ProFlex™ PCR thermal cycler (applied biosystems by life technologies). PCR products were sent to a commercial sequencing company (Inqaba Biotechnical Industries (Pty) Ltd, Pretoria, South Africa) for purification and sequencing in both directions. Resultant sequences were assembled, and chromatogram-based contigs were generated and trimmed using Geneious R11 (http://www.geneious.com) (Kearse et al. 2012). Sequence and species identity were verified against previously published sequences using the Basic Local Alignment Search Tool (BLAST) (Altschul et al. 1990). Sequences obtained in the current study were deposited in the NCBI GenBank database under accession numbers MT925726 (18S rRNA), MT928357 (28S rRNA), MT925727 (16S), and MT920964 (COI).

For the partitioned phylogenetic analysis, representative sequences (18S rDNA, 28S rDNA, 16S rDNA, and COI) from the Cryptopidae, Plutoniumidae, Scolopocryptopidae and Scolopendridae (outgroup) were downloaded from GenBank and aligned to the sequences generated in the current study (Table 1). Concatenated gene sequences were aligned using the Clustal W 2.1 alignment tool (Larkin et al. 2007) under the default settings as implemented in Geneious R11. The final alignment consisted of 27 sequences with a total of 5091 bp positions (1786 bp 18S rDNA, and 2070 bp 28S rDNA, 518 bp 16S rDNA, and 715 bp COI). The partitioned Bayesian inference (BI) analysis was performed using MrBayes 3.2.2 (Huelsenbeck and Ronquist 2001) implemented from within Geneious R11. Prior to the analyses, a model test was performed to determine the most suitable nucleotide substitution model according to the Akaike information criteria (AIC) using jModelTest 2.1.7 (Darriba et al. 2012). The model with the best AIC score for the 18S rRNA and 16S rRNA markers was the General Time Reversible model (Tavaré and Miura 1986) with an estimated proportion of invariable sites and a discrete gamma distribution (GTR + I + G). The model with the best AIC score selected for the 28S rRNA and COI markers was GTR + G. For the BI analysis, the alignment was partitioned according to the 18S rRNA (1–1786 bp), 28S rRNA (1787–3856 bp), 16S rRNA (3857–4375 bp) and COI (4376–5091 bp) genes; the Markov Chain Monte Carlo (MCMC) algorithm was run for 10 million generations, sampling every 100 generations, and using the default parameters. The first 25% of the trees were discarded as ‘burn-in’ with no ‘burn-in’ samples being retained. Results were visualised in Tracer (Rambaut et al. 2018) (implemented from within Geneious R11), to assess convergence and the ‘burn-in’ period.

**Table 1.** List of species and GenBank accession numbers used in the current study.

Family	Species	Country	18S	28Sb	28Sc	16S	COI
Cryptopidae	*Cryptops anomalans*	UK	KF676406	KF676353	–	KF676457	KF676499
	*Cryptops australis*	Australia	AY288692	AY288708	–	AY288723	–
	*Cryptops doriae*	Thailand	KF676407	KF676354	–	KF676458	KF676500
	*Cryptops galatheae*	Argentina	KF676408	KF676355	–	KF676459	KF676501
	*Cryptops hortensis*	UK	JX422708	JX422582	JX422597	JX422684	JX422662
	*Cryptops lamprethus*	New Zealand	JX422709	JX422583	JX422598	JX422685	JX422663
	***Cryptops legagus* sp. nov.**	**Botswana**	**MT925726**	**MT928357**	**MT928357**	**MT925727**	**MT920964**
	*Cryptops niuensis*	Fiji	JX422710	JX422584	JX422599	JX422686	–
	*Cryptops parisi*	UK	KF676409	KF676356	–	KF676460	KF676502
	*Cryptops punicus*	Italy	KF676410	–	–	KF676461	KF676503
	*Cryptops sarasini*	New Caledonia	JX422711	JX422585	JX422600	JX422687	JX422664
	*Cryptops spinipes*	Australia	AY288693	AY288709	–	AY288724	AY288743
	*Cryptops trisulcatus*	Italy	AF000775	AF000783	AF000783	HQ402493	HQ402544
	*Cryptops typhloporus*	South Africa	KF676411	–	–	KF676462	KF676504
	*Cryptops indicus*	Vietnam	KF676412	KF676357	–	KF676463	KF676505
	*Cryptops weberi*	Indonesia	HQ402518	HQ402535	HQ402535	KF676464	HQ402551
Plutoniumidae	*Theatops erythrocephalus*	Portugal	AF000776	HM453279	HM453279	HM453222	–
Scolopocryptopidae	*Newportia quadrimeropus*	Mexico	HQ402511	KF676358	–	HQ402494	HQ402546
	*Newportia divergens*	Guatemala	JX422714	KF676359	–	JX422691	JX422668
	*Newportia ernsti*	Dominican Republic	JX422715	JX422587	–	JX422692	JX422669
	*Newportia monticola*	Costa Rica	HQ402514	KF676360	HQ402531	HQ402497	KF676507
	*Newportia stolli*	Guatemala	JX422719	JX422591	–	JX422696	JX422673
	*Newportia collaris*	Brazil	KF676415	KF676361	–	KF676467	KF676508
	*Scolopocryptops macrodon*	Guyana	JX422721	JX422607	JX422607	JX422699	JX422675
	*Scolopocryptops melanostomus*	Fiji	JX422723	KF676363	JX422609	JX422701	JX422677
	*Scolopocryptops miersii*	Brazil	JX422720	KF676364	JX422606	JX422697	JX422674
Scolopendridae	*Scolopendra morsitans*	Senegal	HQ402519	HQ402537	HQ402537	HQ402501	HQ402553

## Results

### Order Scolopendromorpha Pocock, 1895

**Family Cryptopidae Kohlrausch, 1881**

**Genus *Cryptops* Leach, 1815**

**subgenus Cryptops Leach, 1815**

#### Cryptops (Cryptops) legagus sp. nov.

Taxon classificationAnimaliaScolopendromorphaCryptopidae

27CF3B26-FE9D-5332-A556-28057562BC3E

http://zoobank.org/D0C3D8B8-9EAD-4083-B85A-EB004500D761

**Figure 1.**
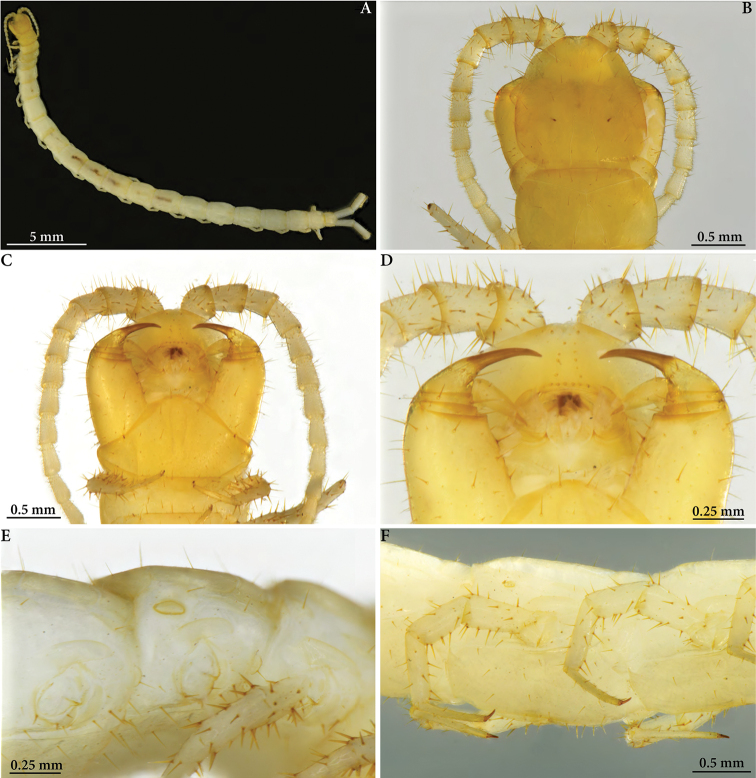
Cryptops (Cryptops) legagus sp. nov., holotype (NHMW 10149) **A** habitus, dorsal view **B** head and T1, dorsal view **C** head and segment 1, ventral view **D** detail of head, ventral view **E** segments 2–4, lateral view, showing spiracle on segment 3 **F** legs 9–10, lateral view.

**Figure 2.**
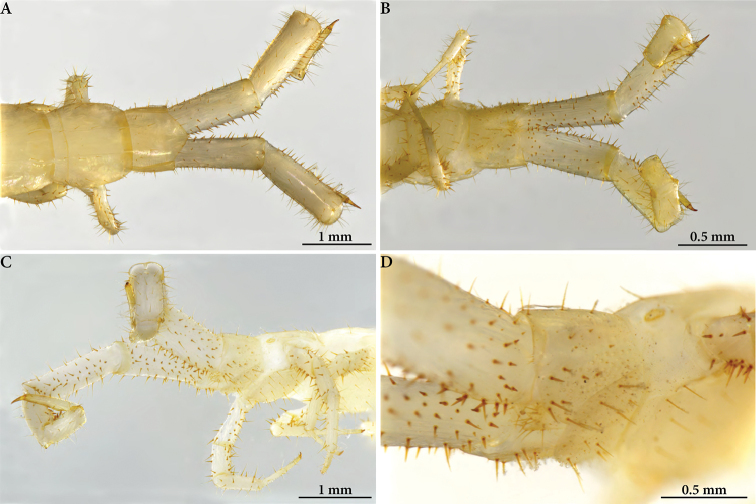
Cryptops (Cryptops) legagus sp. nov., holotype (NHMW 10149). **A–C** segments 19–21, dorsal, ventral and posterolateral views, respectively **D** ultimate leg-bearing segment, ventrolateral view.

**Figure 3.**
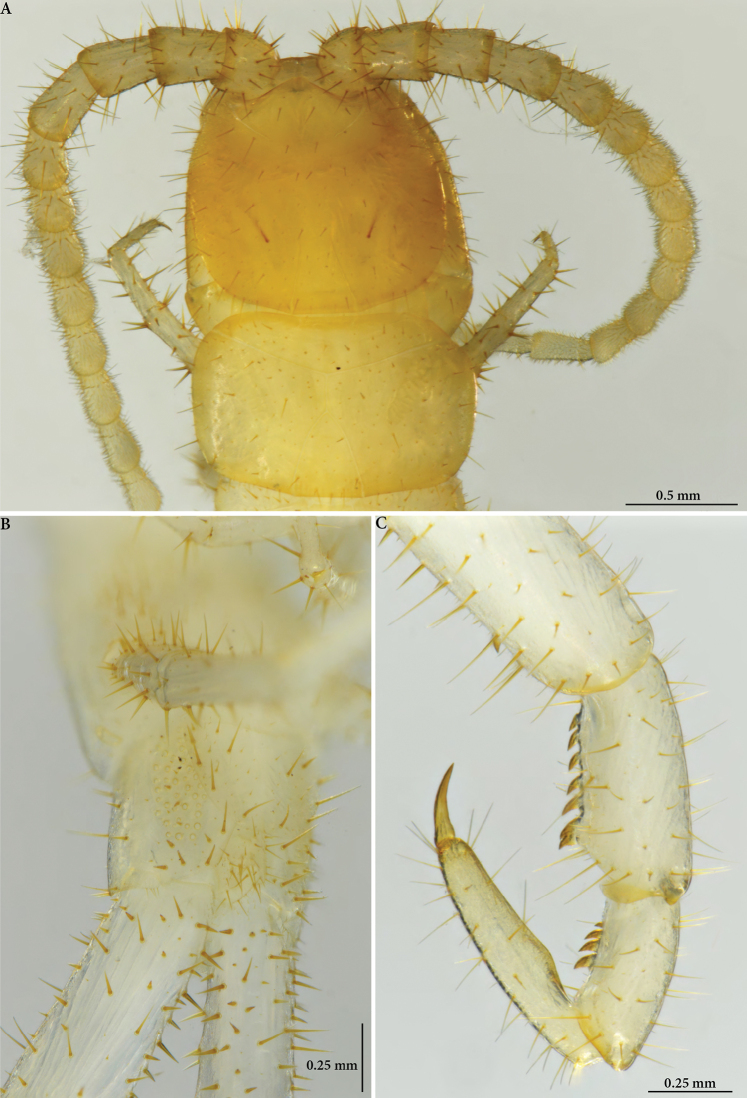
Cryptops (Cryptops) legagus sp. nov., paratype NHMW 10152 **A** head and segment 1, dorsal view **B** ultimate leg-bearing segment, posterolateral view, showing coxopleural pore field **C** distal articles of ultimate leg, showing femoral, tibial and tarsal saw teeth.

**Figure 4.**
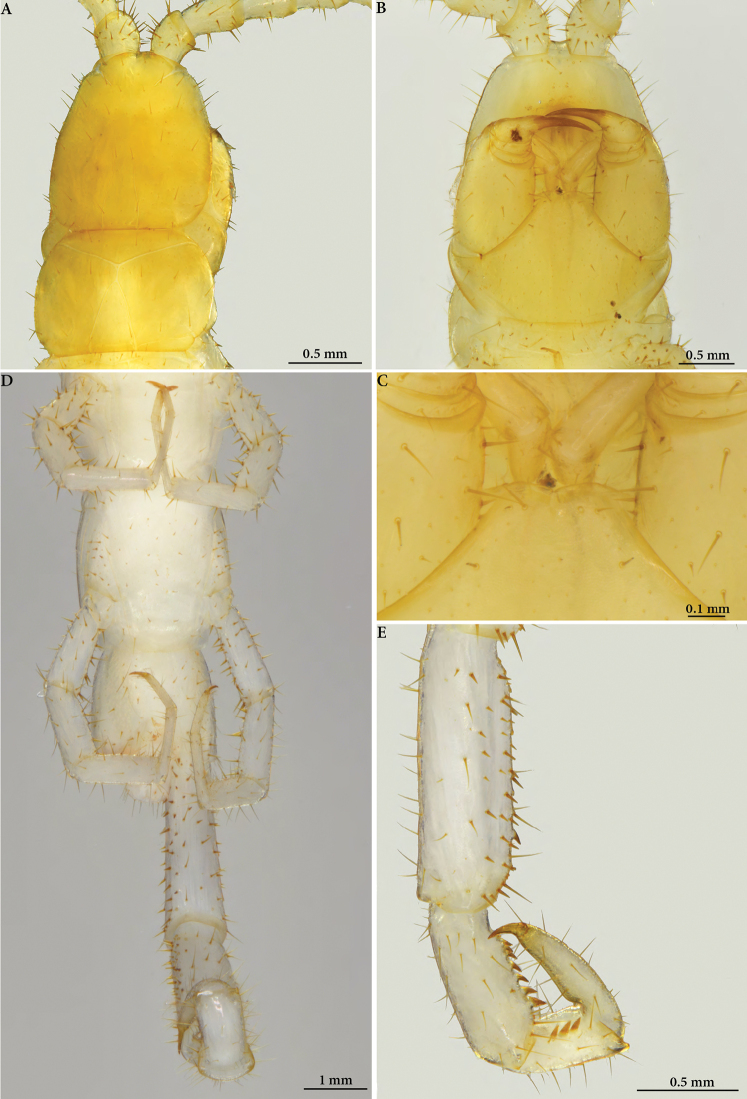
Cryptops (Cryptops) legagus sp. nov., paratype NHMW 10150 **A, B** head and segment 1, dorsal and ventral views **C** forcipular coxosternal margin, ventral view **D** segments 19–21, ventral view **E** distal articles of ultimate leg, showing femoral, tibial and tarsal saw teeth.

**Figure 5.**
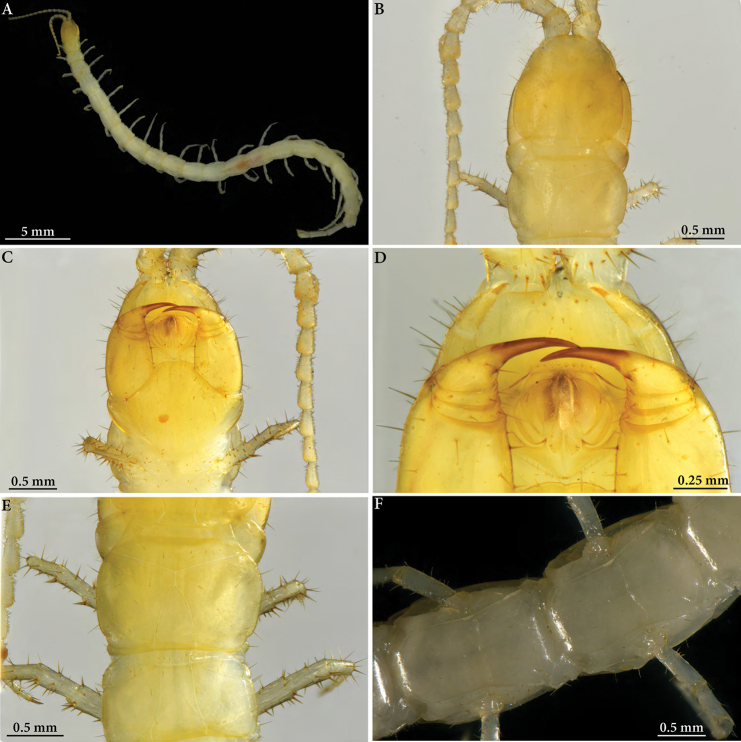
Cryptops (Cryptops) legagus sp. nov., paratype NHMW 10151 **A** habitus, dorsal view **B, C** head and segment 1, dorsal and ventral views **D** detail of head (clypeus, first maxilla and forcipule), ventral view **E** leg-bearing segments 1 and 2, dorsal view **F** cruciform sulci on sternites.

**Figure 6.**
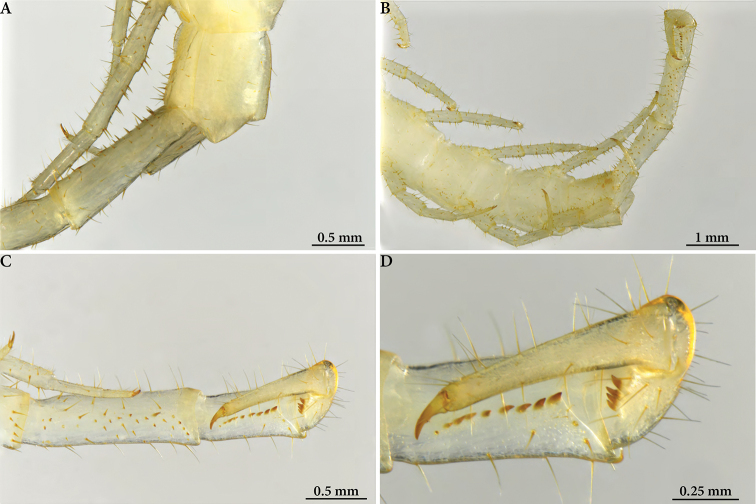
Cryptops (Cryptops) legagus sp. nov., paratype NHMW 10151 **A** segments 20–21, dorsal view **B** segments 18–21, ventrolateral view **C, D** distal articles of ultimate leg and detail of tibia, tarsus and pretarsus, ventral views, showing saw teeth.

Figs 1 , 2 , 3 , 4 , 5 , 6

##### Material.

***Holotype*.**NHMW 10149 (Figs 1–2), Paradise Road Balcony, Diviner’s Cave, Koanaka Hills, 20°8'32.20"S, 21°12'36.60"E, leg. 25.xi.2012, G. Du Preez (see “Habitat”).

***Paratypes*.** All leg. G. Du Preez. NHMW 10150, Diviner’s Cave, leg. 27.iv.2011; NHMW 10151, ‘Calcite Baboon Chamber’, Diviner’s Cave, leg. 27.iv.2011; NHMW 10152, ‘Pirates Cove’, Dimapo Cave (Gcwihaba Hills), leg. 1.v.2013.

##### Diagnosis.

Cephalic plate contacts T1 without consistent overlap by either. Cephalic plate with paramedian sutures on posterior half and short anterolateral sutures. T1 with shallow V-shaped anterior transverse suture, short median suture and diverging curved, diagonal sutures. Paramedian sutures complete from T2. Pretarsal accessory spines elongate, more than half length of claw. Saw teeth on ultimate leg 1 + 6–8 + 3–4.

##### Description.

The following is based on the holotype unless indicated otherwise, with variation in paratypes indicated in square parentheses.

Length (anterior margin of cephalic plate to posterior margin of telson) 28.5 mm [23.0–31.7 mm].

Cephalic plate orange; TT1–2, forcipular segment and basal part of antenna pale orange, other tergites, sternites and legs more yellow.

Paramedian sutures on posterior half of cephalic plate gently sinuous and converging along most of their length, parallel on their anterior part. Anterolateral sutures short, straight. Fine, slender setae relatively sparse on cephalic plate and tergites, most arranged with bilateral symmetry.

Antenna of 17 articles, extending back to anterior part of T4 [posterior half of T3]. Basal 4–4.5 articles scattered with moderately long, pigmented setae; articles 5–10 with longer setae in a whorl around basal part of article, with short, dense setae prevalent; articles 11–17 densely covered with short setae.

Clypeal setae arranged as 2 (+2 small) + 2 + 2 + 2 + 1 + 2 and transverse band of 8 prelabral setae in holotype; paratypes include 2 (+2 small) + 1 + 2 + 2 + 2.

Coxosternal margin biconvex, bearing a short marginal seta and variably a longer submarginal seta on each side. Coxosternum with relatively sparse, symmetrically arranged short setae, more pervasively scattered with minute setae. Tibia but not femur complete on outer side of forcipule.

Both rami of anterior transverse suture on T1 nearly straight, converging to a point medially from which a short median suture extends posteriorly, then branches into divergent sutures with gentle outward convexity. Paramedian sutures complete from TT2–20; sutures on T2 with posterior half more strongly divergent posteriorly than anterior half, more or less bell-shaped, from T3 posteriorly progressively more parallel. Oblique sutures on TT2–3[4]. Lateral crescentic sulci on TT3–19.

Spiracles elongate oval in outline.

Sternites 2–19 with cruciform sulci. Endosternite on anterior segments without trigonal sutures.

Prefemur, femur and tibia on locomotory legs with strongly pigmented setae, many of those of tibia finer than on more proximal articles; tarsus with more slender, paler setae. Tarsal articulations distinct, mostly with negligible flexure on legs 1–18, flexed on legs 19–21 [all tarsi flexed in NHMW 10150]. Pretarsi of legs 1–20 with pair of long accessory spines, consistently more than half length of claw, up to 75% length of claw on some legs; accessory spines lacking on ultimate leg.

Tergite of ultimate leg-bearing segment with two straight sectors on posterior margin that converge medially to a blunt angle; shallow depression posteriorly. Sternite of ultimate leg-bearing segment with lateral margins gently convex outwards, posterior margin nearly straight or gently convex. Coxopleural pore field elongate oval, occupying anterior 75% of coxopleuron, pore-free margin with up to five fairly robust setae arranged as an anterior pair and a posterior row of three. All specimens with more than 30 coxal pores in area not concealed by sternite, ca 60 in highest count, a nearly complete pore field; pores variable in size; two or three short, robust setae and a few more tiny setae within pore field.

Ultimate leg of paratype (body length 25.8 mm) with prefemur 1.4 mm, femur 1.5 mm, tibia 0.9 mm, tarsus 1 0.5 mm, tarsus 2 0.65 mm, pretarsus 0.2 mm. Ultimate leg with distinctly densest and most robust, lanceolate setae on ventromedial parts of prefemur and femur, these articles sparsely setose dorsally. Saw teeth 1 + 6–7[8] + 3–4.

##### Etymology.

*Legaga*, Tswana for “cave”.

## Discussion

As noted in the Introduction, troglobitic species of *Cryptops* are members of either of the subgenera *Cryptops* or *Trigonocryptops*. Most of the apomorphies for *Trigonocryptops* are not present in *C. legagus* sp. nov., and in these characters the species corresponds to the nominate subgenus. Notably, the endosternite is not delimited by trigonal sutures, the clypeus lacks an anterior setose area outlined by sutures, and the femur and tibia of the ultimate legs lack distal spinose projections.

**Figure 7.**
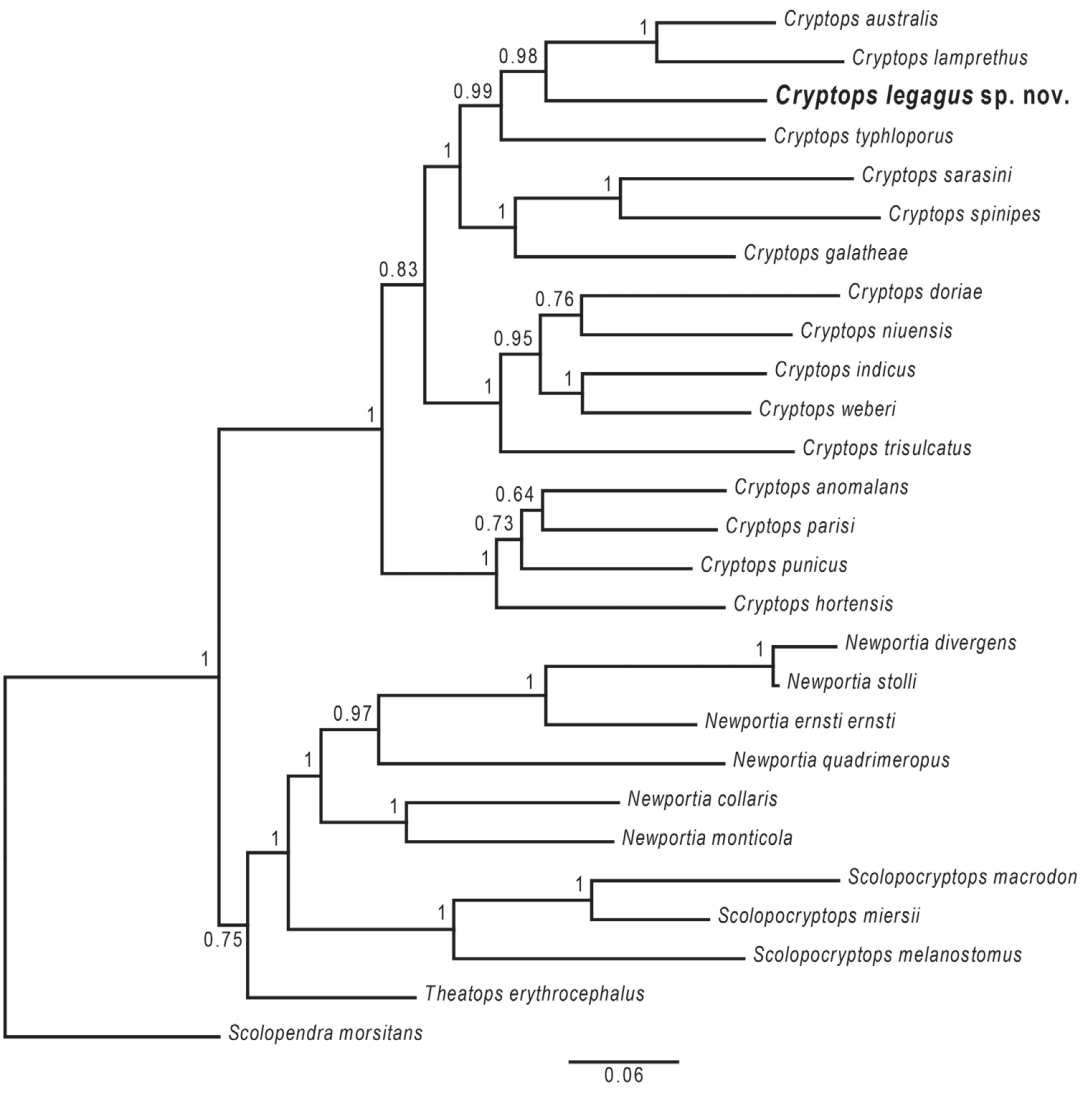
Bayesian tree for blind scolopendromorphs based on partitioned concatenated datasets of four molecular loci 18S rRNA, 28S rRNA, 16S rRNA and cytochrome *c* oxidase subunit I. Numbers at nodes are posterior probabilities. The scale bar represents 0.05 nucleotide substitutions per site.

No species of *Cryptops* shares the observed combination of suture configurations on the cephalic plate and T1. The inverted Y-shaped sutures on T1 are reminiscent of *C. trisulcatus* Brölemann, 1902, and even more so to some specimens of *C. anomalans* Newport, 1844 (such as the synonymous *C. savignyi hirtitarsis* Brölemann; see Brölemann 1930, fig. 340) and a few other taxa of the *C. anomalans* group sensu Lewis (2011). The new species is readily distinguished from *C. trisulcatus* in having a substantially longer median suture on T1 and longer paramedian sutures on the posterior part of the cephalic plate. Our phylogenetic analysis (Fig. 7) does not recover an especially close relationship between *C. legagus* sp. nov. and either *C. trisulcatus* or *C. anomalans*, implying convergence in the shared suture patterns.

The molecular data indicate closest relationships to other Southern Hemisphere species of Cryptops (Cryptops). All four loci independently recover the New Zealand species *C. lamprethus* Chamberlin, 1920 as a close relative, and 16S and COI both find a clade including *C. lamprethus* and *C. typhloporus* Lawrence, 1955 from South Africa. The combined data for all four genes add the New Zealand/Australian *C. australis* Newport, 1845 to this clade, allying it most closely to *C. lamprethus*, with *C. legagus* sp. nov. and *C. typhloporus* as successive sister species. The three related species all lack sutures on the cephalic plate and T1 and are members of the *C. doriae* group within Old World C. (Cryptops) as defined by Lewis (2011). This consists of species having incomplete paramedian sutures on the cephalic plate, lacking an anterior transverse suture on T1, and bearing one or more femoral saw teeth on the ultimate leg. The first and third of these characters are shared by *C. legagus* sp. nov., although the sutures on the cephalic plate are longer in *C. legagus* sp. nov. than in all the others, and the T1 sutures differ strikingly. As relationships within this Southern temperate clade are strongly supported in the molecular tree (posterior probability 0.98–1 for all three nodes), as is a closer affinity between it and C. (Trigonocryptops) than to the nominate species of the *C. doriae* group, at least some of the characters delimiting groups morphologically are evidently homoplastic.

Despite its troglobitic occurrence, only the relatively pale pigmentation and elongate pretarsal accessory spines (shared with troglomorphic Australian *Cryptops*: Edgecombe 2005, 2006) suggest a degree of troglomorphy. Neither the antennae nor legs show much elongation, nor are the tergites/sternites conspicuously longer than in typical epigean species, nor are numbers of saw teeth on the ultimate legs particularly high. The slight troglomorphic modifications suggest that it is unlikely to be an epigean species.

## Supplementary Material

XML Treatment for Cryptops (Cryptops) legagus
